# Tracking unlabeled cancer cells imaged with low resolution in wide migration chambers via U-NET class-1 probability (pseudofluorescence)

**DOI:** 10.1186/s13036-022-00321-9

**Published:** 2023-01-24

**Authors:** Paola Antonello, Diego Morone, Edisa Pirani, Mariagrazia Uguccioni, Marcus Thelen, Rolf Krause, Diego Ulisse Pizzagalli

**Affiliations:** 1 Università della Svizzera italiana, Faculty of Biomedical Sciences, Institute for Research in Biomedicine, CH-6500 Bellinzona, Switzerland; 2 Graduate School of Cellular and Molecular Sciences, University of Bern, CH-3012 Bern, Switzerland; 3 Università della Svizzera italiana, Euler institute, CH-6962 Lugano-Viganello, Switzerland; 4 FernUni, Faculty of Mathematics and Informatics, Brig, Switzerland

**Keywords:** Cell tracking, Brightfield microscopy, Cell migration

## Abstract

Cell migration is a pivotal biological process, whose dysregulation is found in many diseases including inflammation and cancer. Advances in microscopy technologies allow now to study cell migration in vitro, within engineered microenvironments that resemble in vivo conditions. However, to capture an entire 3D migration chamber for extended periods of time and with high temporal resolution, images are generally acquired with low resolution, which poses a challenge for data analysis. Indeed, cell detection and tracking are hampered due to the large pixel size (i.e., cell diameter down to 2 pixels), the possible low signal-to-noise ratio, and distortions in the cell shape due to changes in the z-axis position. Although fluorescent staining can be used to facilitate cell detection, it may alter cell behavior and it may suffer from fluorescence loss over time (photobleaching).

Here we describe a protocol that employs an established deep learning method (U-NET), to specifically convert transmitted light (TL) signal from unlabeled cells imaged with low resolution to a fluorescent-like signal (class 1 probability). We demonstrate its application to study cancer cell migration, obtaining a significant improvement in tracking accuracy, while not suffering from photobleaching. This is reflected in the possibility of tracking cells for three-fold longer periods of time. To facilitate the application of the protocol we provide WID-U, an open-source plugin for FIJI and Imaris imaging software, the training dataset used in this paper, and the code to train the network for custom experimental settings.

**Supplementary Information:**

The online version contains supplementary material available at 10.1186/s13036-022-00321-9.

## Introduction

The regulation of many biological processes is mediated by the migration of cells from one anatomical location to another to exert their function. For example, primordial germ cell migration in zebrafish is essential to ensure the correct organ development [1]. Moreover, the correct development of proper immune responses requires a fine-tuned regulation of leukocyte trafficking and migration [2–4].

Amongst the mechanisms involved in cell migration, chemotaxis polarizes cells and controls the direction of migration toward favorable locations [5, 6]. Hence, several studies focus on the knowledge of the molecular mechanisms and signaling pathways that regulate chemotaxis in vitro and in vivo. However, the directional movement of cells is regulated not only by the type of soluble cues diffused into and retained by the environment but also by the environment itself [7, 8].

**Fig. 1:**
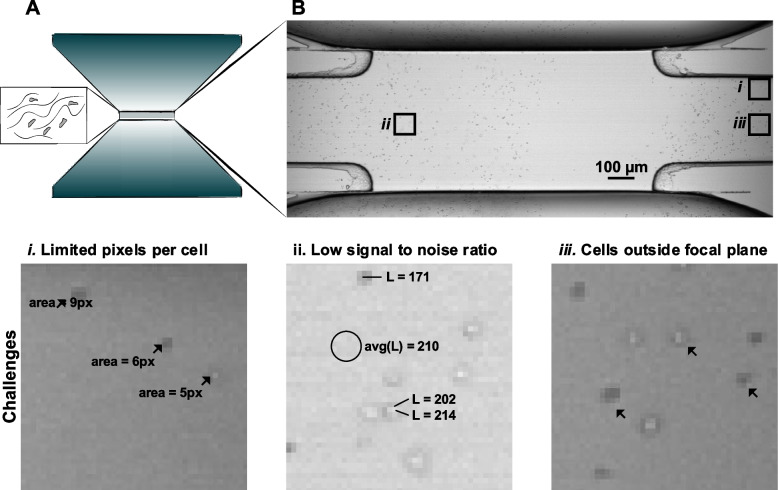
Widefield microscopy in wide 3D migration chambers. **(A).** Representation of a wide migration chamber for chemotaxis assays. **(B).** Widefield image of VAL cells scattered in the field of view using TL. ***i-iii*** challenges for the automatic analysis: (*i*) large pixel size associated with a limited number of pixels per cell, (*ii*) appearance of cells similar to the background associated with a low signal to noise ratio, and (*iii*) different appearance of cells according to the position along the z-axis with respect to the focal plane

Therefore, engineered microenvironments are essential to study cell migration in vitro. Amongst these, 3D migration is a setting where cells are embedded in collagen-like fibers to mimic the extracellular matrix (ECM) *in vitro* [6, 9] (Fig. 1A). Widefield Microscopy (WM) is an established technique to perform long-term imaging studies in large migration chambers. This technique can be applied by recording the fluorescence intensity or the intensity of the light transmitted through the sample, without necessarily requiring fluorescent staining. In this imaging modality, the acquired data consists of a series of 2D grayscale images captured over time.

However, when WM is applied to study the migration of motile cells in large 3D migration chambers, the analysis of the acquired series of images presents specific challenges. Indeed, the classical analysis pipeline involves three steps: cell detection, cell tracking, and computation of motility measures [10, 11]. The application of such a pipeline is hampered at the first step, due to drastic changes in cell shape that introduce cell detection errors. These changes are associated with the frequent squeezing of the cytoskeleton while migrating through dense ECM [6], or introduced as an artifact during the migration along the z-axis. In the last case, cells are imaged outside the focal plane, leading to blurred and enlarged shapes in the acquired images.

Additionally, depending on the experimental settings, cells can require a long period to exert a directional movement. Hence, long acquisition times are needed. Long-time acquisitions may prevent the usage of fluorescent staining (used to facilitate cell detection) due to photobleaching or phototoxicity. Hence, imaging of unlabeled cells using transmitted light (TL) is necessary. Lastly, analysis of cells following long tracks (i.e., > 150 μm), demands a large field of acquisition and necessitates low magnification (i.e. 4X objective). Therefore, the resolution is another challenge for cell detection and subsequently compromises tracking (Fig. 1B).

Recent advances in artificial intelligence methods applied to bioimage analysis remarkably improved the accuracy of cell detection and subsequently tracking [12–14]. Amongst these, end-to-end neuronal networks with convolutional layers such as the U-NET [15] and its variants that transform an input image into another image as output, improved the segmentation of complex structures with respect to single pixel classifiers [16], gaining application in both biomedical imaging for cell detection, counting, and morphological analysis [17, 18]. The usage of U-NET was also demonstrated to improve cell and tracking due to the increased robustness of object detection on binary masks rather than on original images which may suffer from non-uniform illumination or poor signal to noise ratio [19–22].

Although U-NET was applied to many different imaging modalities and cell types, a pipeline to specifically analyze time-lapse images of unlabeled cells acquired with low magnification via brightfield microscopy in 3D migration chambers is still missing.

Therefore, we propose WID-U (U-NET for WIDe migration chambers), a plugin for common bioimaging software such as Imaris (Oxford instruments) and FIJI, that converts the TL signal from brightfield microscopy into a fluorescent like signal (pseudofluorescence) corresponding to the class 1 probability from the U-NET. The signal generated by WID-U yielded an efficient detection of the cells using standard spot-detection methods available in TrackMate [23, 24] and Imaris, which subsequently improved cell tracking accuracy in images with low resolution from 3D in vitro environments.

## Results

### Pipeline to convert TL to pseudo-fluorescence

**Fig. 2:**
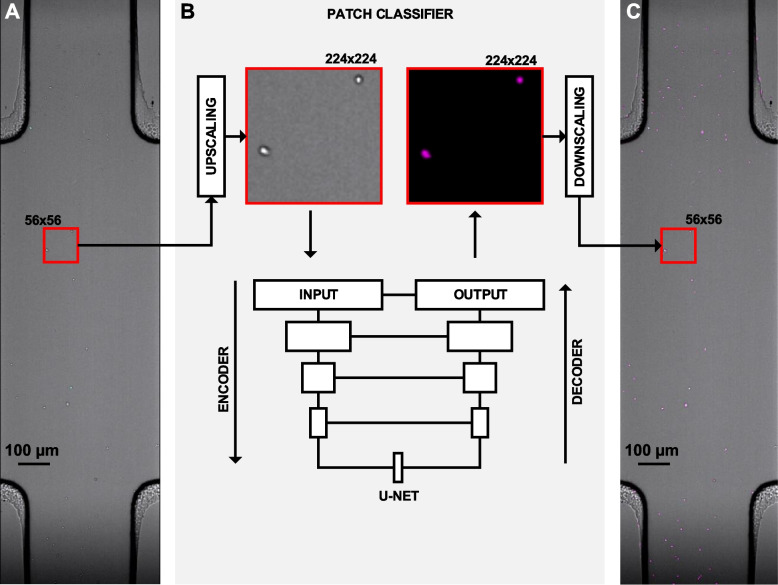
Pipeline to convert TL to pseudofluorescence. **(A).** TL image obtained by widefield microscopy. The red square represents a sliding window of 56 × 56 pixels used for image processing. **(B).** Patch classifier, which employs a U-NET architecture with five fully convolutional layers. An upscaled window (224px x 224px) is used as input. The class-one probability is used to generate a pseudofluorescent image as output (magenta colored). **(C).** The output is then downscaled and combined with the original imaging data to create a virtual imaging channel with pseudo-fluorescence (magenta)

To convert the TL signal from unlabeled cells to pseudo-fluorescence, we developed an image processing pipeline based on deep learning. Such a pipeline is specifically developed to face the challenges arising when images of cells are acquired in large 3D migration chambers, at low magnification (4x) and large fields of view (2 mm × 2 mm). To account for such low magnification and large fields of view, images are processed with a sliding window of 56 × 56 pixels (~ 92 μm × 92 μm) (Fig. 2A, red square). Subsequently, each window is upscaled by a factor of 4 to 224 × 224 pixels, and processed via a patch classifier based on the U-NET architecture [17]. (Fig. 2B). Such architecture receives as input the upscaled TL images (Fig. 2B, grayscale image), and generates as output an image where the intensity of each pixel is the class-1 probability, or pseudofluorescence (Fig. 2B, magenta-colored image). The output of the U-NET is then downscaled and reassembled as a new imaging channel of the original image (Fig. 2C). To train the network, datasets consisting of upscaled image pairs were manually created using a custom tool that virtually zooms-in on selected areas of different videos and at different time points. This allowed examples of cells lying at different focal planes, and in areas with different illumination, conferring to the trained network robustness to brightness/contrast changes (Fig. S1).

### Enhanced cell detection and tracking of B cell lymphoma in 3d migration chambers

**Fig. 3:**
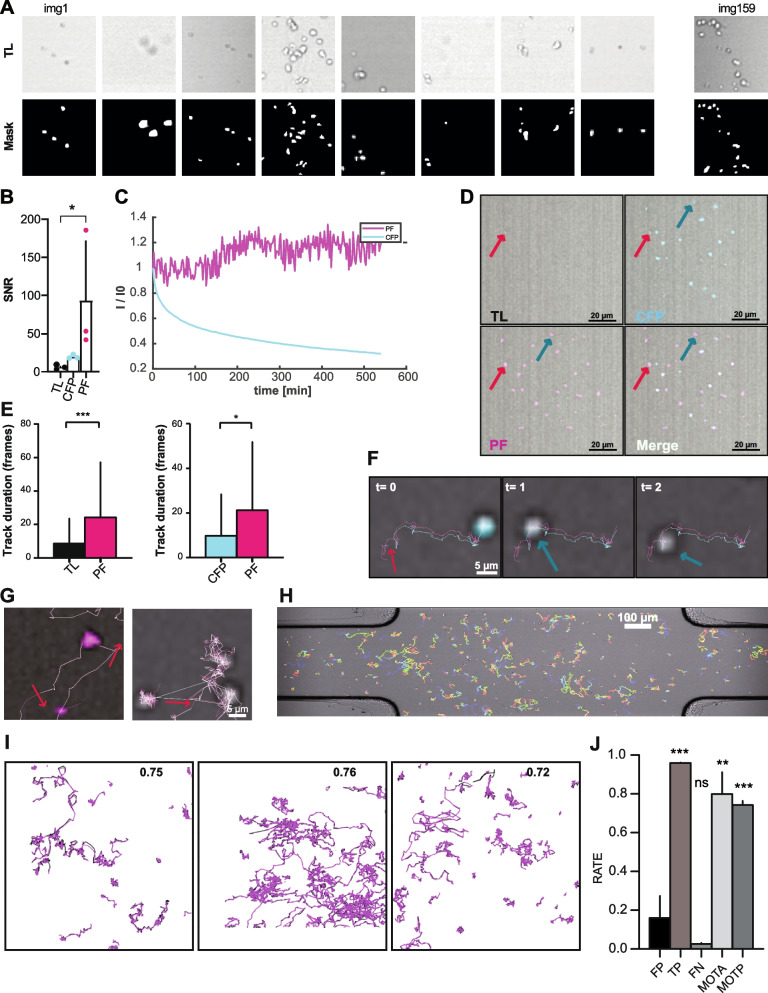
Enhanced cell tracking of VAL cells in 3d migration chambers using pseudofluorescence. **(A).** Representative image pairs from the dataset used for training, including TL images (up), and manually annotated binary masks (bottom) (n of images included = 159). **(B).** Comparison of the signal-to-noise ratio (SNR) between cells observed via TL, CFP labeled cells (CFP), and pseudofluorescence (PF). The SNR value of each population derives from the mean of three independent observations. **(C)**. Comparison of the intensity variation (intensity at time t / intensity at time 0, average of all the cells) over time of pseudofluorescence (magenta) and CFP (blue), showing the effect of photobleaching on CFP. **(D).** Representative micrographs showing the transformation into pseudofluorescence of cells which are poorly visible in TL and outside the focal plane. **(E).** Comparison of automatic tracking accuracy (Track duration) using TL, pseudo-fluorescence, CFP-labeled cells and pseudofluoresence calculated on the CFP positive cells. Values of each population were calculated from the mean of three independent observations. Track duration is expressed in % w.r.t the total video duration. **(F).** Representative micrographs showing tracks of cells obtained using PF (magenta lines) and CFP signals (cyan lines). Red arrow indicates a cell not detected using CFP due to photobleaching. **(G).** Representative micrograph showing tracking errors (red arrow, glitch between cells in close proximity) when using PF with respect to TL. **(H).** Results obtained on a 3D migration chamber (color-coded tracks, blue = 0 s, red = 8 hours). **(I).** Visual comparison between manually tracked cells (black lines), and automatically tracked cells using pseudofluorescence channel (magenta lines). num. Tracks = 40, 96, 59. **(J).** Quantitative evaluation of multiple object tracking accuracy. Statistics performed with Mann-Whitney t-test relative to FP as control, * = P < 0.05 *** = P < 0.001

We applied the proposed pipeline to analyze videos of VAL cells (a Germinal Center-derived B cell lymphoma), acquired in 3D microenvironments. A dataset of 150 image pairs was used to train the network (Fig. 3A). Then, we compared the quality of the pseudofluorescence signal with respect to TL, or real fluorescence emitted by cyan fluorescent protein positive (CFP+) cells. The proposed pipeline yielded a significant improvement in the signal-to-noise ratio (SNR) with respect to TL images (Fig. 3B), and a higher but not significantly improvement in SNR with respect to real fluorescence (CFP). In contrast to CFP, the intensity of the pseudo-fluorescent signal did not suffer from photobleaching (Fig. 3C). Despite noise was introduced by the automatic adjustment of focal plane at each time point, the mean intensity of the pseudo-fluorescent cells never decreased below 80% of the intensity at the initial time point.

Moreover, the proposed pipeline increased the visibility of cells, which were out of focus or with deformed shapes (Fig. 3D). Altogether, these properties make pseudofluorescence similar to real fluorescence, but with increased stability over time.

To validate the effect of pseudofluorescence on the quality of cell tracking, we performed automatic cell tracking using TL, real fluorescence (CFP+ cells), or pseudofluorescence signals. Pseudofluorescence yielded significantly more accurate tracks than the original TL signal, with an average three-fold increase in the track duration (Fig. 3E). In comparison with real fluorescence, track duration was longer, especially in the late time points when the fluorescent signal was fading (Fig. 3B-E). In general, pseudofluorescence decreased the number of tracking errors, resulting in fewer interrupted tracks (Fig. 3F) and fewer glitches when cells were in close proximity (Fig. 3G), facilitating the automatic tracking of cells using pseudofluorescence in large 3D microenvironments (Fig. 3H, Fig. S2).

Additionally, we manually tracked 195 cells from three different experiments corresponding to 23,991 spots (all the cells in the field of view were tracked, respectively 40, 96, 59 tracks, and 5182, 10,117, 8692 spots) and compared these tracks with the ones obtained by applying the proposed pipeline (Fig. 3I). The true positive rate of spot detection was greater than 95%, while the false positive rate was lower than 17% and a false negative rate lower than 5%. To evaluate the accuracy of cell tracking instead (i.e. penalizing track switch errors), we performed a multi-object tracking analysis (MOTA) [25] obtaining a MOTP score greater than 0.74 (Fig. 3J).

### Enhanced cell detection and tracking of MDA-MB231 breast cancer cells with heterogeneous shapes

**Fig. 4:**
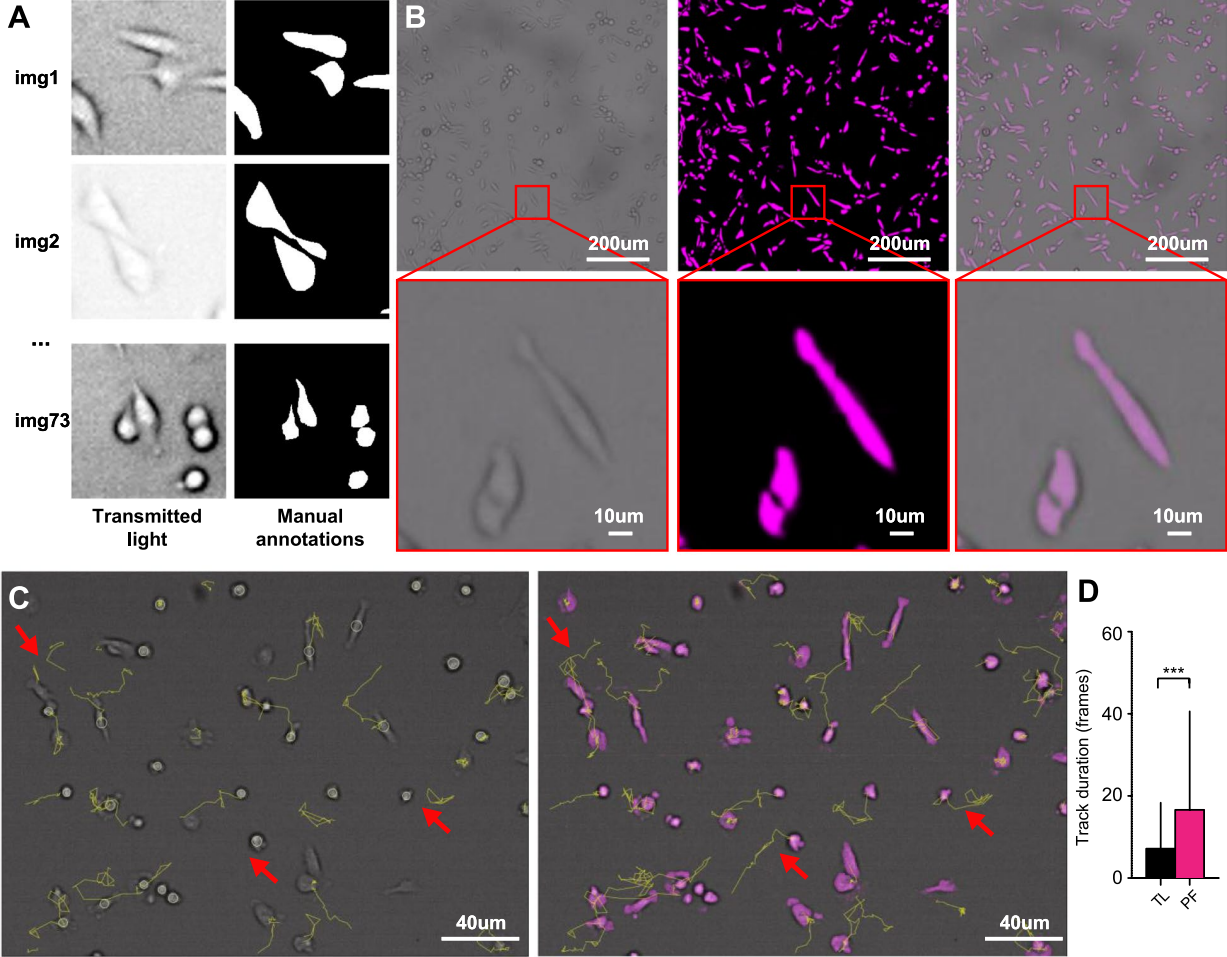
Enhanced tracking of MDA-MB-231 breast cancer cells with heterogeneous shapes **(A)**. Representative micrographs of the image pairs used to train the network. **(B)**. Micrographs representing images of cells with heterogeneous shapes (round, or thin-elongated) in transmitted light, pseudofluorescence, and merged. **(C)**. Comparison between tracks obtained using transmitted light (left) or pseudofluorescence (right). Arrows indicate tracking errors obtained using the transmitted light signal only. **(D)**. Comparison in track duration using transmitted light vs pseudofluorescence signal with the proposed protocol. *n* = 76 cells (TL), 80 cells (PF). Statistics performed with Mann-Whitney t-test, *** = P < 0.001

To validate the applicability of the pipeline to track cell types with substantially different morphologies, we performed chemotaxis assays using MDA-MB-231, a human breast cancer cell line established from a pleural effusion of a 51-year-old Caucasian female with metastatic mammary adenocarcinoma. These are epithelial adherent cancer cells with a heterogeneous morphology, either spindle-shaped (long and thin) or rounded. A dataset with 73 image pairs from 3 independent experiments depicting cells with both morphologies was generated (Fig. 4A). Then, the network was re-trained and applied to convert the TL signal to pseudofluorescence. The computed class-1 probability was used as input to a tracking algorithm based on threshold detection and linking, obtaining significantly longer tracks with respect to the ones obtained using the raw TL signal (Fig. 4B-C).

### WID-U plugin

**Fig. 5:**
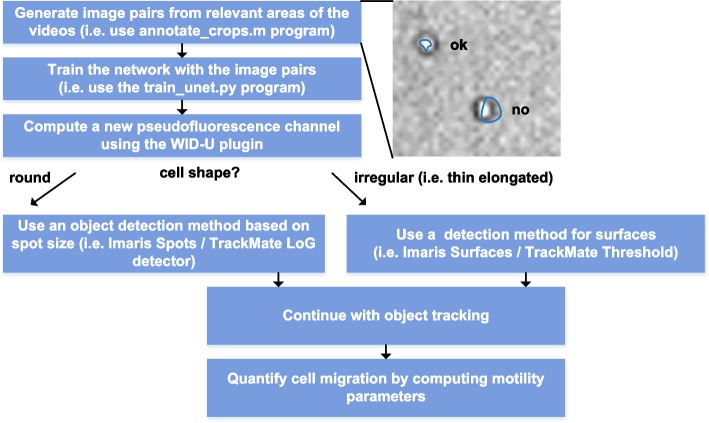
Usage workflow. Summary of the protocol to follow to enhance cell tracking using WID-U

To facilitate the execution of the pipeline for TL to fluorescence conversion, a plugin for the Imaris (Oxford instruments), and a plugin for FIJI bioimaging software have been developed. The protocol that makes use of the plugin to enhance cell tracking is summarized in (Fig. 5). Briefly, it will be sufficient to load the image sequence in the preferred imaging software, then launch the WID-U plugin to transform the TL signal into a new channel with pseudofluorescence. Finally, automated cell tracking can be performed on the generated new channel. Regarding the installation, the software comes with three different parts: the plugin itself, the program to re-train, if needed, the network for custom cell types, and the program that performs the computations using deep learning. These last two programs can be either installed on the same machine where FIJI/Imaris is installed or configured on a remote machine dedicated to computation (a CUDA-enabled machine is recommended to speed up the execution). To configure the connection to such a machine, it will be sufficient to configure the IP address in the plugin (Fig. S3). Instructions are included in the README file. Additionally, to use WID-U without a GPU-enabled machine, we made available a macro to export the images, process them on a remote machine (i.e. , a free online deep learning platform such as Google COLAB), and a macro to import the results in the desired software (Fig. S3).

## Materials and methods

### Cell line and cultures

VAL cells were cultured in RPMI-1640 medium supplemented with 10% heat-inactivated fetal bovine serum (FBS), 1% Penicillin/Streptomycin, 1% GlutaMAX, 1% NEAA, 1% sodium-pyruvate, and 50 μM β-mercaptoethanol. CFP+ cells were cloned as described previously [26]. MDA-MB-231 cells were cultured in Dulbecco’s Modified Eagle Medium (DMEM) containing D-glucose 4.5 g L1, and glutaMAX (619650–026, GIBCO, ThermoFisher Scientific, Switzerland) supplemented with fetal bovine serum 10% (16000–044, GIBCO, ThermoFisher), and penicillin-streptomycin 1% (15,070,063, GIBCO, ThermoFisher Scientific). Cells were incubated under standard culture conditions (CO2 5%, O2 95%, 37 °C)**.**

### Migration assays

3D migration of VAL cells was performed using the 3D chamber μ-Slide from Ibidi as described by *Antonello* et al. 10.3389/fimmu.2022.1067885. Briefly, cells were embedded in a collagen matrix formed by 1.6 mg/mL PureCol (Collagen, Sigma-Aldrich), 0.36% PBS supplemented with 0.36% FBS, 0.036% P/S, 1.5 μg/mL recombinant human ICAM-1/CD54 Fc chimera (R&D systems) at 4 °C. The temperature was slowly raised over 45 min to 37 °C to induce a homogeneous collagen fiber polymerization. Complete medium was added to both side reservoirs. After 24 hours, 15 μL of 400 nM CXCL12 were added to one of the reservoirs and time-lapse video microscopy was performed for 6 hours at 20 seconds time intervals using an ImageXpress® (Molecular Devices) high throughput microscope, equipped with an incubation system set to CO_2_ 5%, O_2_ 95%, 37 °C, with a Nikon Plan Apo 4x / NA 0.2 and 20 mm working distance objective. CFP was excited with a Lumencore LED lamp, excitation band 438/24, collection band 483/32. For brightfield imaging, the condenser was set to Koehler illumination. Collection was performed with an Andor Zyla sCMOS camera. Migration assays of MDA-MB-231 cells were performed using the μ-Slide chemotaxis chambers from Ibidi, according to the manufacturer’s instructions. Briefly, cells were resuspended at 3 × 10^6^ cells ml^− 1^ in Dulbecco’s Modified Eagle Medium (DMEM) and glutaMAX (619650–026, GIBCO, ThermoFisher Scientific, Switzerland) supplemented with fetal bovine serum 1% (16000–044, GIBCO, ThermoFisher). The observation area of the chamber was filled with cells, and the chamber was placed in the incubator (CO_2_ 5%, O_2_ 95%, 37 °C) for 1 h to allow cell adherence. Time-lapse video microscopy images were recorded for 18 h with a time interval of 600 seconds using the ImageXpress as described above.

### Transformation of TL images into pseudofluorescence

TL images were converted into pseudofluorescence by employing an end-to-end neural network with convolutional layers based on the U-NET architecture [17]. A dataset with pairs of TL and pseudofluorescence (binary masks) was created manually drawing the contour of cells. Such dataset included 150 images of 56 × 56 pixels (91 × 91 μm) from different experiments. These images were upscaled to 224 × 224 pixels to facilitate annotation then downscaled to 112 × 112 pixels for training and augmented to 15′000 images. The trained network was then applied to convert images of size > = 1000 × 500 pixels, to pseudofluorescence by classifying a moving window of 56 × 56 pixels. The class-1 probability computed by the U-NET was used as pseudofluorescence. Hyperparameters on training can be found in the code in Supplementary material. Briefly, Adam optimizer, loss = binary cross-entropy, initial learning rate = 10^− 4^, batch size = 2, epochs = 60. Data augmentation was performed using Keras image generators, with rotation range = 0.5, zoom range = 0.5, vertical and horizontal shift = 0.5, shear range = 0.2, horizontal and vertical flip, and zero filling.

### Cell tracking

Initially, TL images were transformed into pseudofluorescence images. Then, cells were detected and tracked using the Spots tracking functionality of the Imaris software (Oxford Instruments, v.7.7.2) in the original TL channel, in the imaging channel capturing fluorescence, and in the pseudofluorescence channel. In all cases, an estimated spot diameter of 8 um was selected and background subtraction was enabled to account for non-uniform illumination. Tracking was performed using an autoregressive motion model, with a maximum distance of 20 μm, and a maximum gap size of 0. Tracks shorter than 300 seconds were excluded from the analysis. Tracks were divided into two classes (WT and CFP+ cells), based on the mean fluorescence intensity of the imaging channel centered on CFP. Finally, the duration of each track was computed. Tracks outside the migration channel were deleted manually.

Automatic tracking was also performed using TrackMate in FIJI [23] using the LoG spot detector or automatic thresholding, and Simple Lap tracker for spot tracking. The same threshold described before for the tracking in Imaris were used also for the tracking in TrackMate.

### Image and tracking measures

Track measures were exported from Imaris or TrackMate and processed in Matlab to compute track duration, fluorescence decay and SNR. SNR was estimated as [avg (FG) – avg. (BG)] / std. (BG) where FG is the intensity of the pixels in the foreground and BG pixels in the background as previously described [12].

### Statistics

SNR and Track duration values were analyzed with PRISM software. Statistics performed with ONE WAY ANOVA * *P* < 0.05, **P < 0.01 ****P < 0.0001.

## Discussion

The application of deep learning to in vitro time-lapse imaging improved the tracking accuracy of cancer cells in large 3D migration microenvironments. In this paper, this was achieved by training a U-NET architecture with a custom dataset for B cell lymphoma (globular shapes) and a custom dataset for breast cancer cells (heterogeneous shapes). To apply our pipeline to other cell types or other imaging modalities, the network can be re-trained. In the cases included in this paper, 70 to 150 image pairs with data augmentation were sufficient to improve tracking accuracy. However, to enhance the robustness of cell detection, image pairs included in the training set should be representative of cells in different areas of the microenvironment. The creation of the training set was facilitated by upscaling. This is associated with an increased precision during the manual annotation when cells had a small area (i.e., 4 pixels), and was completed by an imaging expert in less than 6 hours for both cases. However, to minimize the number of training examples required, we recommend the usage of transfer learning approaches [17].

The tracking was improved mainly as a consequence of more accurate spot detection. Several tools based on supervised machine learning are nowadays available for cell segmentation. However, to track cell centroids over time, accurate shape reconstruction may not be required. Indeed, the class-1 probability generated by the U-NET, which decreases towards the borders of the cells (Fig. S4), demonstrated particularly appropriate for the subsequent application with a variety of spot detection methods as previously demonstrated [19–22], and allows the user to select more or less detected objects by defining an intensity threshold, in line with the most common tracking pipelines. In this study, we tested two approaches typically used in bioimaging: watershed with background subtraction (Imaris) and LoG detector (FIJI/TrackMate). In both cases, performances improved. Performances increased also when automatic thresholding (FIJI/TrackMate) was used for spot detection. This suggests that pseudofluorescence is uniform across the field of view and spot detection based on intensity becomes possible. Our protocol facilitates the computation of this signal systematically, either automatically sending data from FIJI/Imaris to a cluster with a GPU or allowing export/import data to be processed on free deep learning resources such as Google COLAB.

The multi-object tracking analysis revealed a MOTP score of 0.74 when a simple LAP tracking algorithm was used [23]. This suggests that the method can be applied to automatize the analysis, despite further improvements can be obtained by more advanced linking algorithms, or manual post-correction. To further enhance accuracy, pseudofluorescence can be used in combination with recently developed methods for cell detection, such as those based on geometric properties and deep learning [27].

In conclusion, the proposed pipeline allowed cell tracking in large 3D migration chambers over extended periods of time, retaining the simplicity of cell detection as when real fluorescence is used, but avoiding photobleaching and other side effects of cell labeling.

## Supplementary Information

**Additional file 1: Supplementary Fig. 1.** Benchmark in response to intensity shifts. A. representative micrographs of images with shifted signal excursion (minimum) from 0 to 232. B. Segmentation accuracy metrics vs. intensity shifts. IoU refers to Intersection over Union. Pixel difference is the mean error in pixel intensity vs. manually annotated binary masks. *n* = 4 different image series, with 32 different levels.

**Additional file 2:** **Supplementary Fig. 2.** Representative micrograph showing tracks of cells obtained with PF (magenta square), TL (black square), or CFP (cyan square), using Trackmate analysis. Detection of cells was performed using either LogDetector (above) or Threshold detector (below)

**Additional file 3: Supplementary Fig. 3.** Usage workflow. The pipeline to convert low-resolution TL images to pseudofluorescence is made available via the WID-U plugin. For sporadic uses it is possible to export the images and process them on an online deep learning platform such as Google COLAB (top). For routine uses the tool can be easily installed on a GPU-enabled machine or on same computer by using a virtual machine. In Imaris, once installed and configured for communication with a deep learning-enabled machine, the user can launch the plugin and use the Spots/Surfaces tools for cell detection/tracking. In FIJI, once the image has been opened, the user can launch the WID-U Plugin. It will ask for the IP address of the machine to be used for the computation. Once completed, a new imaging channel will be created and the TrackMate plugin can be used for spot detection and tracking

**Additional file 4: Supplementary Fig. 4. **Class-1 probability decay. Representative map of the class-1 probability showing peaks inside the objects

## Data Availability

Data and code are released under the Open Source GPL v3 license at https://github.com/pizzagalli-du/wid-u The datasets used to train the U-NET architecture (crops and binary masks) are available as PNG files in training_data_VAL.zip and training_data_MDA-MB-231.zip for VAL lymphoma and MDA-MB-231 breast cancer cells respectively. We also provide the weights of the pre-trained U-Net models for VAL and MDA-MB-231 cells observed with a pixel size of 1.62 um (UNET_VAL.h5, and UNET_MDA-MB-231.h5). The U-NET model was defined and trained in the Keras (v 2.3.1) framework, on a TensorFlow-GPU (v 2.2.0) backend, using Python (v 3.8.3). the script to re-train the network with custom dataset is available in the file “train_unet.py”, while the program to create the training dataset with upscaling and manually drawing binary masks was written in Matlab r2019b and is available in the file “annotate_crops.m”. Raw microscopy data are deposited on the IMMUNEMAP Open Data platform (www.immunemap.org).
