# Supplementary figures and images for: Tracking unlabeled cancer cells imaged with low resolution in wide migration chambers via U-NET class-1 probability (pseudofluorescence)
Source: J Biol Eng. 2023 Jan 24;17:5. doi: 10.1186/s13036-022-00321-9 (PMC9872392; doi:10.1186/s13036-022-00321-9)

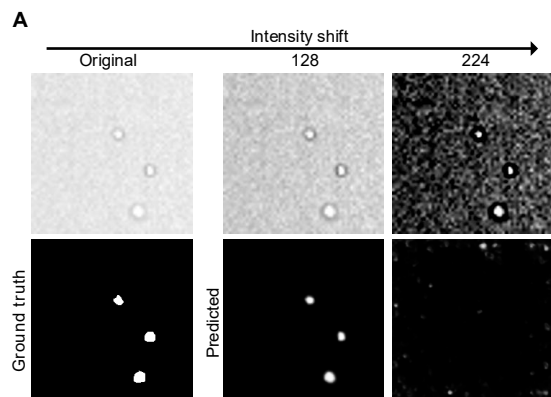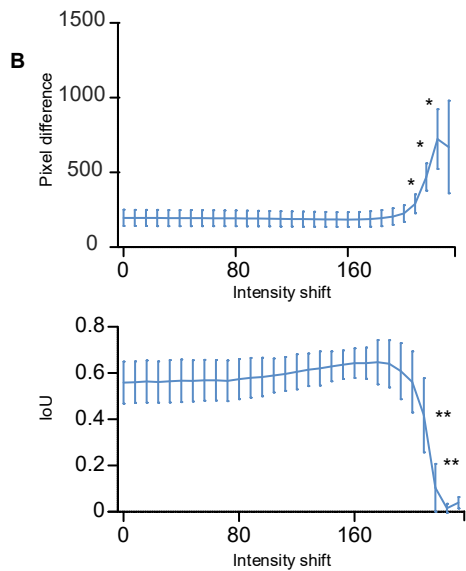

Supplement: Supplementary file 1 — Additional file 1: Supplementary Fig. 1. Benchmark in response to intensity shifts. A. representative micrographs of images with shifted signal excursion (minimum) from 0 to 232. B. Segmentation accuracy metrics vs. intensity shifts. IoU refers to Intersection over Union. Pixel difference is the mean error in pixel intensity vs. manually annotated binary masks. n = 4 different image series, with 32 different levels. [file 13036_2022_321_MOESM1_ESM.pdf]

A

PF

TL

CFP

Supplementary figure 2

Log Detector

Threshold Detector

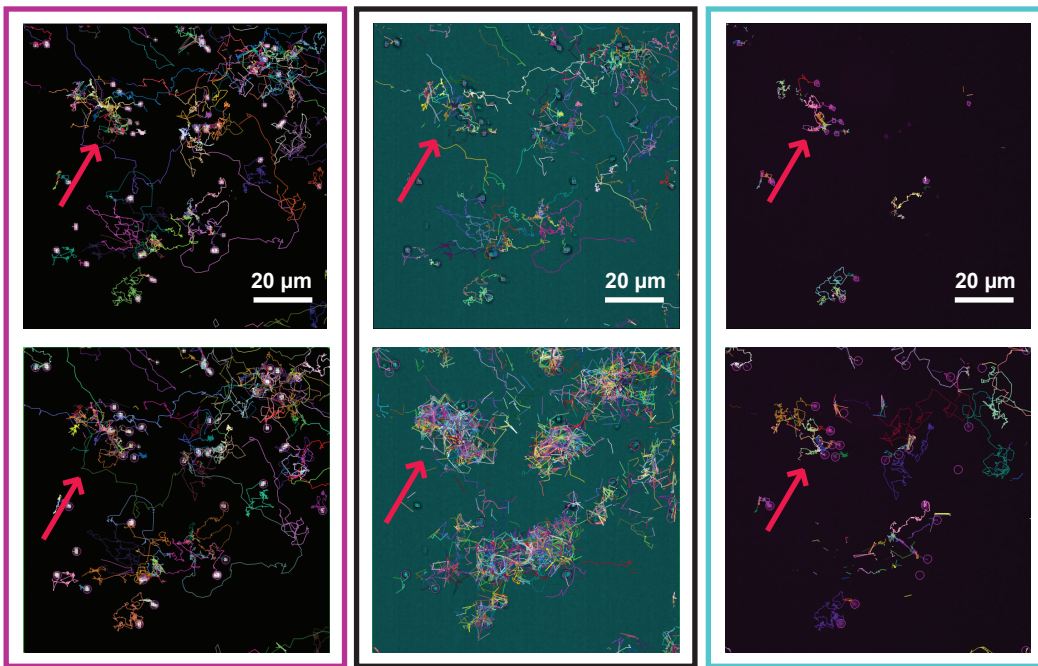

Supplement: Supplementary file 2 — Additional file 2: Supplementary Fig. 2. Representative micrograph showing tracks of cells obtained with PF (magenta square), TL (black square), or CFP (cyan square), using Trackmate analysis. Detection of cells was performed using either LogDetector (above) or Threshold detector (below) [file 13036_2022_321_MOESM2_ESM.pdf]

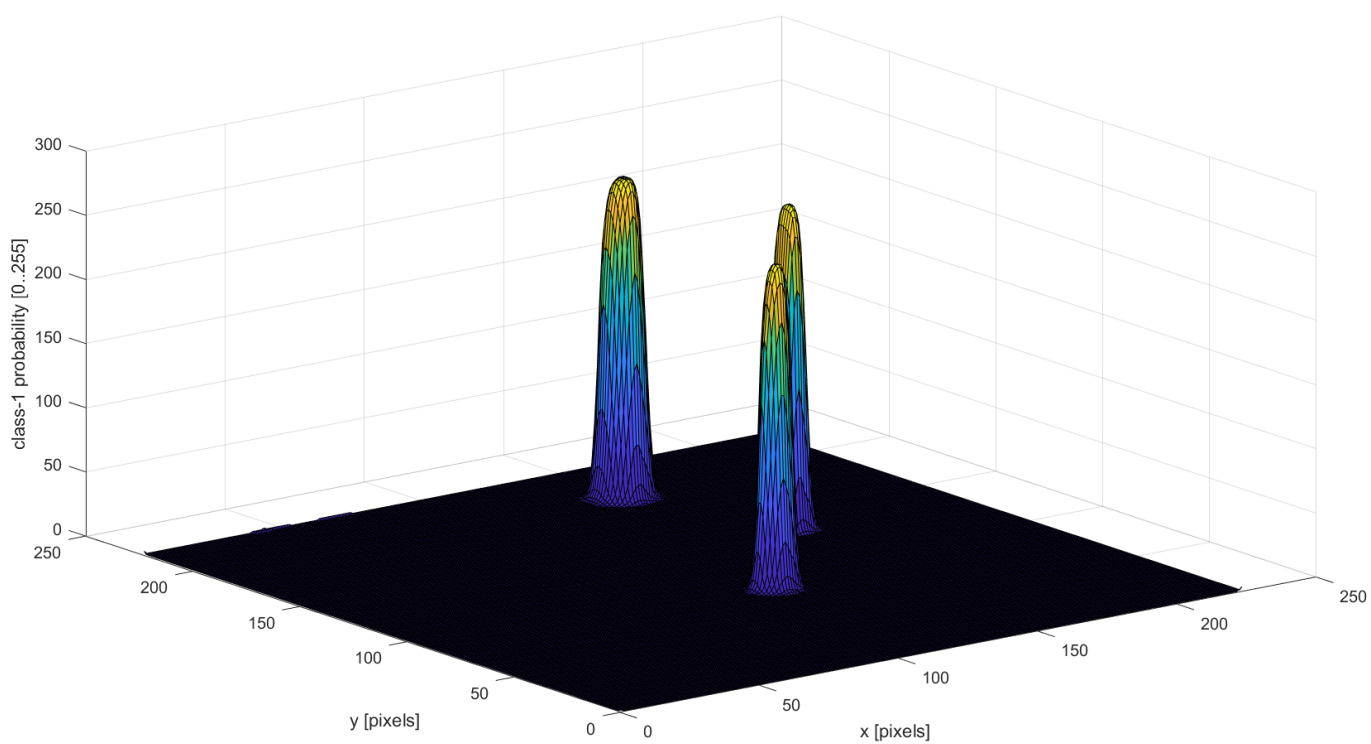

Supplement: Supplementary file 4 — Additional file 4: Supplementary Fig. 4. Class-1 probability decay. Representative map of the class-1 probability showing peaks inside the objects [file 13036_2022_321_MOESM4_ESM.pdf]
