# Supplementary material for: Tracking unlabeled cancer cells imaged with low resolution in wide migration chambers via U-NET class-1 probability (pseudofluorescence)
Source: J Biol Eng. 2023 Jan 24;17:5. doi: 10.1186/s13036-022-00321-9 (PMC9872392; doi:10.1186/s13036-022-00321-9)
Supplement: Supplementary file 3 — Additional file 3: Supplementary Fig. 3. Usage workflow. The pipeline to convert low-resolution TL images to pseudofluorescence is made available via the WID-U plugin. For sporadic uses it is possible to export the images and process them on an online deep learning platform such as Google COLAB (top). For routine uses the tool can be easily installed on a GPU-enabled machine or on same computer by using a virtual machine. In Imaris, once installed and configured for communication with a deep learning-enabled machine, the user can launch the plugin and use the Spots/Surfaces tools for cell detection/tracking. In FIJI, once the image has been opened, the user can launch the WID-U Plugin. It will ask for the IP address of the machine to be used for the computation. Once completed, a new imaging channel will be created and the TrackMate plugin can be used for spot detection and tracking [file 13036_2022_321_MOESM3_ESM.pdf]

CASE 1: USE A FREE ONLINE DEEP LEARNING PLATFORM

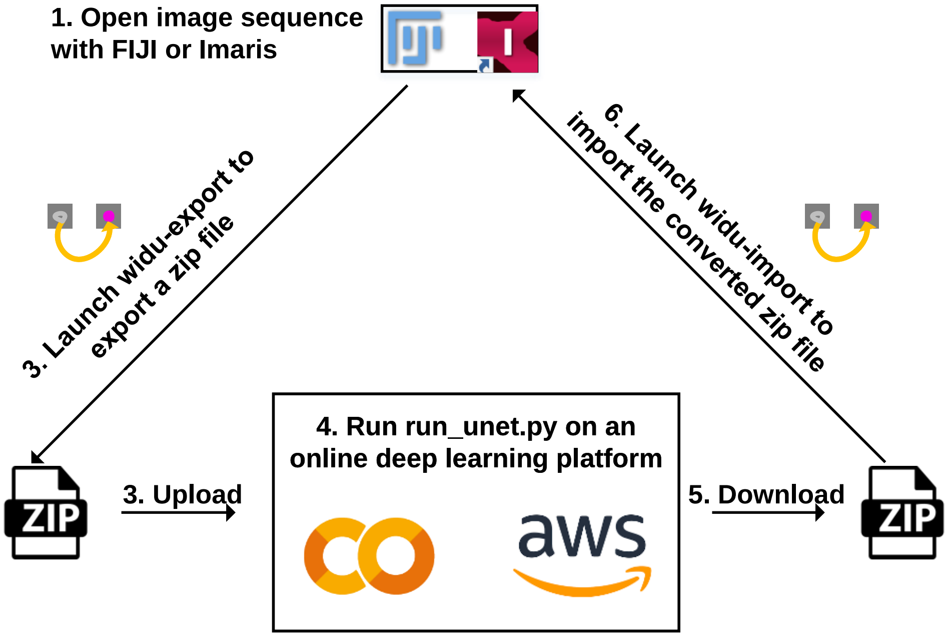

CASE 2: USE A GPU-ENABLED DEEP LEARNING MACHINE (recommended for intensive use)

- Install WID-U server on a GPU-enabled machine and WID-U plugin on the personal computer

IMARIS XTension	FIJI Plugin
1. Open image sequence with Imaris	1. Open image sequence with FIJI
↓	↓
2. Launch the WID-U plugin	2. Launch the WID-U plugin

- Enter the parameters to connect to WID-U server

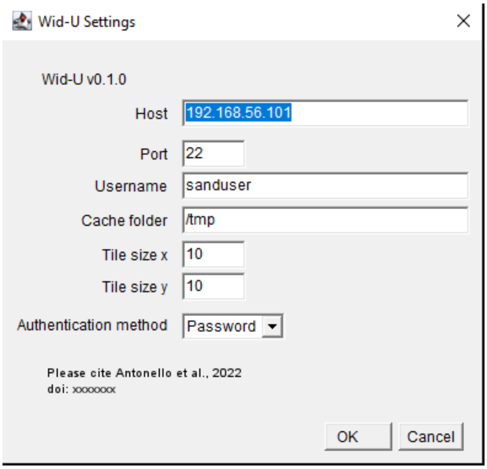

Wid-U Settings

Wid-U v0.1.0

Host: 192.168.56.101

Port: 22

Username: sanduser

Cache folder: /tmp

Tile size x: 10

Tile size y: 10

Authentication method: Password

Please cite Antonello et al., 2022
doi: xxxxxxx

OK | Cancel
